# The Men Who Have Sex with Men HIV Care Cascade in Rio de Janeiro, Brazil

**DOI:** 10.1371/journal.pone.0157309

**Published:** 2016-06-14

**Authors:** Rodolfo Castro, Marcelo Ribeiro-Alves, Renato Girade Corrêa, Monica Derrico, Katia Lemos, Jose Roberto Grangeiro, Beto de Jesus, Denise Pires, Valdilea G. Veloso, Beatriz Grinsztejn

**Affiliations:** 1 Instituto Nacional de Infectologia Evandro Chagas, Fundação Oswaldo Cruz, Rio de Janeiro, RJ, Brazil; 2 Instituto de Saúde Coletiva, Universidade Federal do Estado do Rio de Janeiro, Rio de Janeiro, RJ, Brazil; 3 Departamento de DST, Aids e Hepatites Virais, Ministério da Saúde, Brasília, DF, Brazil; 4 Gerência de DST, Aids, Sangue e Hemoderivados, Secretaria de Estado de Saúde, Rio de Janeiro, RJ, Brazil; 5 Grupo Arco-Íris, Rio de Janeiro, RJ, Brazil; 6 Associação Internacional de Lésbicas, Gays, Bissexuais, Transexuais e Intersexuais para a América Latina e o Caribe, Buenos Aires, CABA, Argentina; University of Athens, Medical School, GREECE

## Abstract

Brazil has a concentrated HIV epidemic and men who have sex with men (MSM) are disproportionately affected. Yet, no data is available on the HIV care cascade for this population. This study aimed to assess the HIV care cascade among MSM newly diagnosed through innovative testing strategies in Rio de Janeiro. Data from 793 MSM and travestites/transgender women (transwomen) tested for HIV at a non-governmental LGBT organization and a mobile testing unit located at a gay friendly venue were analyzed. A 12-month-after-HIV-diagnosis-censored cohort was established using CD4, viral load and combination antiretroviral therapy (cART) longitudinal data from those diagnosed with HIV. A cross-sectional HIV care cascade was built using this data. The relative risks of achieving each cascade-stage were estimated using generalized linear models according to age, self-declared skin-color, education, history of sexually transmitted diseases (STD), drug use and prior HIV testing. From Jan-2013 to Jan-2014, 793 MSM and transwomen were tested, 131 (16.5%) were HIV-infected. As of January 2015, 95 (72.5%) were linked to HIV care, 90 (68.7%) were retained in HIV care, 80 (61.1%) were on cART, and 50 (38.2%) were virally suppressed one year after HIV diagnosis. Being non-white (Relative risk [lower bound; upper bound of 95% confidence interval] = 1.709 [1.145; 2.549]) and having a prior HIV-test (1.954 [1.278; 2.986]) were associated with an HIV-positive diagnosis. A higher linkage (2.603 [1.091; 6.211]) and retention in care (4.510 [1.880; 10.822]) were observed among those who were older than 30 years of age. Using community-based testing strategies, we were able to access a high-risk MSM population and a small sample of transwomen. Despite universal care coverage and the test-and-treat policy adopted in Brazil, the MSM cascade of care indicates that strategies to increase linkage to care and prompt cART initiation targeted to these populations are critically needed. Interventions targeting non-white and young MSM should be prioritized.

## Introduction

Globally, the HIV epidemic among men who have sex with men (MSM) and transgender women (TGW) are showing expanding trajectories [1,2]. Most of the new HIV cases in Latin America occur among these populations [3]. In Brazil, while the HIV prevalence in the general population is 0.6%, among MSM it is 14.2% [4], reinforcing that this population, especially the very young MSM [5], is disproportionately affected by HIV infection in the country [6].

Although TGW represent smaller population than MSM, they have extremely elevated HIV infection rates. A meta-analysis of data from 15 countries estimated an HIV prevalence of 17.7% (95% CI 15.6–19.8) in this population, with an odds ratio of 50.0 (95% CI 26.5–94.3) for HIV infection among TGW versus all adults of reproductive age in low- and middle-income countries [7].

In Brazil, HIV testing at no cost is available in public health care centers as well as in 383 voluntary counseling and testing (VCT) units across the country. Nevertheless, access to testing and services is markedly insufficient, especially for the most vulnerable population groups [8,9]. Studies have shown that about half of Brazilian MSM report prior HIV testing [4,10,11] with only 19% reporting HIV testing in the previous year [12]. Infrequent testing remains a barrier for maintaining accurate HIV serostatus awareness even among those who have previously been tested [13].

Routine HIV testing is critical for timely HIV diagnosis as it triggers the cascade of care for those who are HIV-positive while also providing an unique opportunity for risk management counseling for those who are HIV-negative, including referral for pre-exposure prophylaxis (PrEP) for those at higher risk of HIV acquisition [14]. For vulnerable groups such as MSM and TGW, diverse testing venues are needed to encourage those still unaware of their HIV status to seek testing. In this scenario, community-based testing using HIV rapid tests has been shown to be a valuable option [15].

As part of the activities to guarantee, expand and improve quality and access to sexually transmitted diseases (STD) and HIV prevention services in Brazil, in 2008, the pilot project called, *Quero Fazer* (Let’s Do It) funded by the United States Agency for International Development (USAID) was implemented by the non-governmental organization (NGO) *Espaço de Prevenção e Atenção Humanizada* (EPAH), in partnership with the Brazilian Ministry of Health (BMOH) and five STD/AIDS State programs (including Rio de Janeiro). The *Quero Fazer* Project offered free confidential community-based testing using HIV rapid tests to MSM and transvestites/TGW (herein called transwomen) in stigma free environments. A mobile unit operated by a local NGO and a testing outlet based in a local Lesbian, Gay, Bisexual and Transgender (LGBT) NGO were implemented at each of the 5 Brazilian States. Differently from the regular health services in Rio de Janeiro, these units operated after regular working hours. This study aims to assess the HIV care cascade among MSM and transwomen newly diagnosed with HIV infection through the *Quero Fazer* Project in Rio de Janeiro, Brazil.

## Methods

This analysis was performed using data from the *Quero Fazer* Project in Rio de Janeiro, one of the epicenters of the Brazilian HIV epidemic. In Rio de Janeiro, community-based out of health-care testing using HIV rapid tests were offered in two venues: 1) once a week, on Wednesday evenings, at a mobile testing unit located in front of a unique popular LGBT venue; 2) four days a week, from Monday to Thursday, after working hours, at the site of *Arco-Íris* Group, a well-known LGBT NGO. Potential participants were offered voluntary counseling and testing through trained peers and counselors. All MSM and transwomen tested at both venues during the study period (from January 2013 to January 2014) were included in the analysis. Data were analyzed anonymously with participants’ records/information de-identified prior to analysis. The research was approved with waiver of informed consent by the Instituto de Pesquisa Clínica Evandro Chagas Institutional Review Board (CAEE: 40010014.0.0000.5262).

Data collected at HIV testing sites included: date and venue of the test; date of birth; age; city of residence; state of residence; gender; sexual orientation; skin color; level of education; history of sexually transmitted diseases (STD) in the prior 12 months; substance use in the prior 12 months; prior HIV testing; if previously test in NGO; date of receipt of the test result; and HIV test result.

Time-dependent CD4 cell counts, HIV viral load (VL) results, cART regimens with date of drug dispensation were provided by the BMOH from the national databases, which contain all information related to antiretroviral drug dispensation and CD4 and viral load monitoring. The final dataset was processed censoring all individual information (that is, all CD4, VL and cART dispensation information) 12 months after the date of the HIV diagnosis.

A cross-sectional cascade of HIV care was built including the following stages: HIV-diagnosed (HIV-infected at testing); linked to HIV care (at least one record of CD4 or VL or dispensation of antiretroviral drugs); retained in HIV care (at least two records >90 days apart of CD4 or VL or dispensation of antiretroviral drugs); on cART (at least one record of dispensation of antiretroviral drugs); and virally suppressed (at least on viral load ≤ 50 copies).

We used generalized linear models with logit link function in R software version 3.1.1 [16] to estimate the relative risk and 95% confidence intervals (RR [CI95%]) of reaching a particular stage of the cascade among those MSM who were in the predecessor cascade-stage. Variables explored in regression models as independent predictors of reaching a particular stage of the cascade included age, race/ethnicity, education and history of: STD, drug use and HIV trials. The final model included all variables found to independently predict each of the outcomes. Adjusted risk ratios were given for each covariate while adjusting for the variables found to independently predict each outcome.

## Results

**Fig 1:**
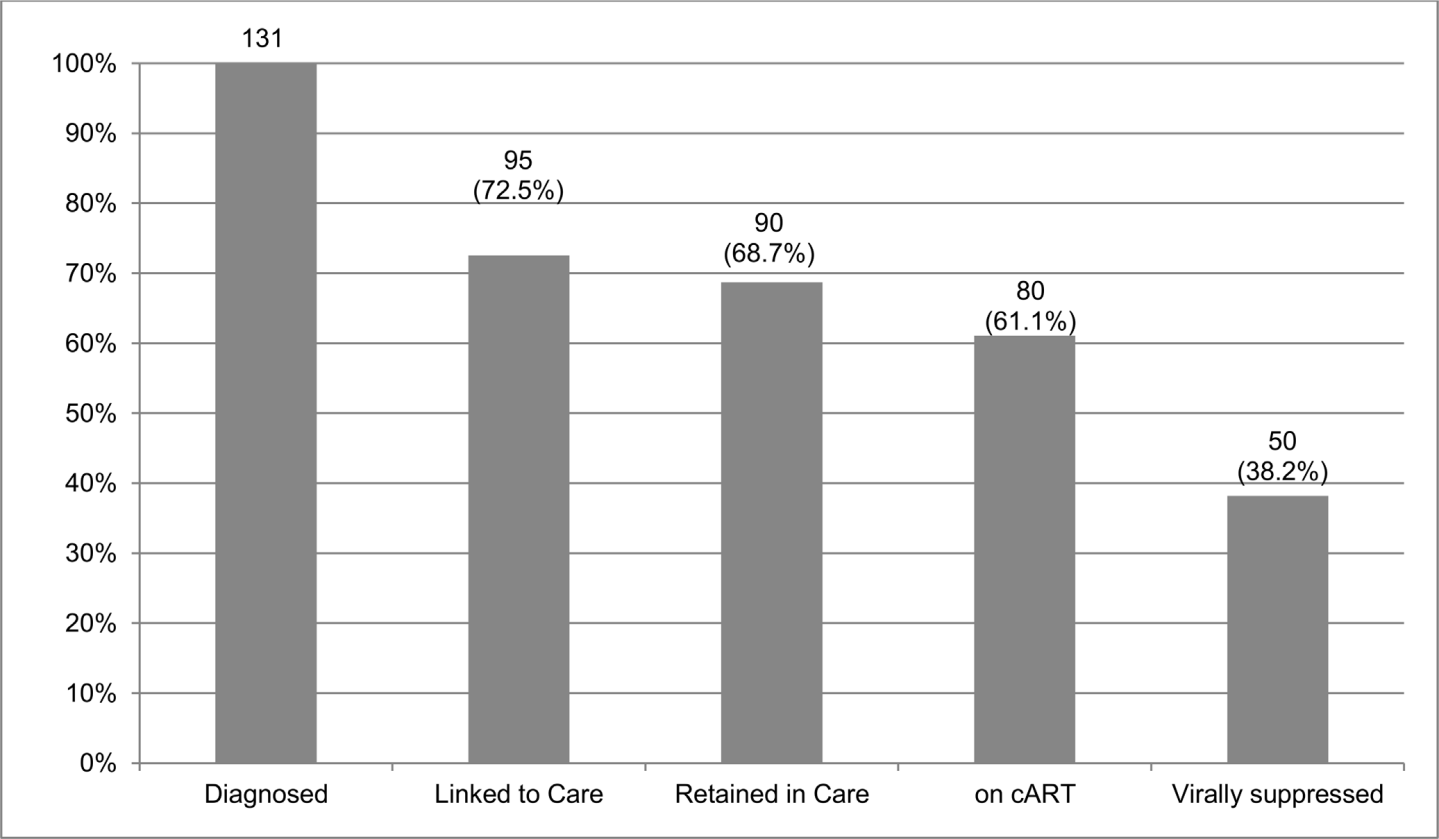
Cascade of HIV care for Brazilian MSM in Rio de Janeiro, Brazil. Results expressed in number and percentage of all HIV-diagnosed MSM for each cascade-stage.

From January 2013 to January 2014, 756 MSM and 37 transwomen were tested (Table 1), 214 (27.0%) in the mobile testing unit and 579 (73.0%) at *Arco-Íris* Group. Overall, 131 (16.5%) were HIV-infected, 123 MSM (16.3% prevalence) and 8 transwomen (21.6% prevalence). This difference in the prevalence for MSM and transwomen was not statistically significant (p = 0.53 [Chi squared]). Forty-three (32.8%) were HIV-diagnosed at the mobile unit, while 88 (67.2%) were diagnosed at *Arco-Íris* Group. Mean age for the HIV-infected MSM and transwomen were 28.1 and 29.3, respectively. For those diagnosed with HIV infection, baseline mean CD4 cell count and viral load were 493 cells/mm3 (CI 95% 443–542) and 77.793 copies/ml (CI 95% 31.116–124.429). As of January 2015, 95 (72.5%) participants were linked to HIV care, 90 (68.7%) were retained in care, 80 (61.1%) were on cART, and 50 (38.2%) were virally suppressed (Fig 1).

**Table 1:** Baseline characteristics of all tested individuals disaggregated by gender identity (MSM and transwomen), Rio de Janeiro, Brazil.

		MSM (n = 756)*			Transwomen (n = 37)*			p-value**	
		HIV-	HIV+	All MSM	HIV-	HIV+	All transwomen	HIV- vs. HIV+	MSM vs. transwomen
Age	<30-year-old	378 (59.72)	72 (58.54)	450 (59.52)	20 (68.97)	7 (87.5)	27 (72.97)	1.000	0.122
	> = 30-year-old	255 (40.28)	51 (41.46)	306 (40.48)	9 (31.03)	1 (12.5)	10 (27.03)		
Skin Color	White	283 (44.71)	41 (33.33)	324 (42.86)	11 (37.93)	2 (25)	13 (35.14)	0.029	0.006
	Non-white	349 (55.13)	82 (66.67)	431 (57.01)	16 (55.17)	6 (75)	22 (59.46)		
	Unknown	1 (0.16)	0 (0)	1 (0.13)	2 (6.90)	0 (0)	2 (5.41)		
Education	High School or Less	273 (43.13)	61 (49.59)	334 (44.18)	25 (86.21)	7 (87.5)	32 (86.49)	0.325	<0.001
	Some College or Higher	355 (56.08)	62 (50.41)	417 (55.16)	4 (13.79)	1 (12.5)	5 (13.51)		
	Unknown	5 (0.79)	0 (0)	5 (0.66)	0 (0)	0 (0)	0 (0)		
Exposure to HIV	Sexual intercourse	626 (98.89)	123 (100)	749 (99.07)	29 (100)	8 (100)	37 (100)	1.000	1.000
	Others	3 (0.47)	0 (0)	3 (0.40)	0 (0)	0 (0)	0 (0)		
	Unknown	4 (0.63)	0 (0)	4 (0.53)	0 (0)	0 (0)	0 (0)		
STD (12 m Hist.)	No	567 (89.57)	105 (85.37)	672 (88.89)	24 (82.76)	5 (62.5)	29 (78.38)	0.099	0.064
	Yes	66 (10.43)	18 (14.63)	84 (11.11)	5 (17.24)	3 (37.5)	8 (21.62)		
Drug use (12 m Hist.)	No	550 (86.89)	111 (90.24)	661 (87.43)	16 (55.17)	3 (37.5)	19 (51.35)	0.913	<0.001
	Yes	78 (12.32)	11 (8.94)	89 (11.77)	12 (41.38)	5 (62.5)	17 (45.95)		
	Unknown	5 (0.79)	1 (0.81)	6 (0.79)	1 (3.45)	0 (0)	1 (2.70)		
History of HIV test	Previously untested	514 (81.20)	87 (70.73)	601 (79.50)	23 (79.31)	4 (50)	27 (72.97)	0.004	0.405
	Previously tested	119 (18.80)	36 (29.27)	155 (20.50)	6 (20.69)	4 (50)	10 (27.03)		
Baseline measures***	Mean CD4	-	493.12 450.44; 538.50]	-	-	490.83 [448.52; 536.41]****	-	-	0.691
	Mean Viral Load	-	76645.21 [76103.34; 77189.56]	-	-	93662.00 [93063.12; 94263.78]****	-	-	0.255

* results are shown as: number (% of all MSM or transwomen)

** Fisher’s exact test was used for all categorical variables and Wilcoxon’s for numeric

*** mean [Poisson CI 95%] for baseline CD4 and VL, available only for patients retained in HIV care

**** only 6 HIV+ transwomen individuals were retained in HIV care.

Being non-white (1.709 [1.145; 2.549]) and having a prior HIV-test (1.954 [1.278; 2.986]) were associated with HIV-positive diagnosis (Table 1). A higher linkage (2.603 [1.091; 6.211] and retention in care (4.510 [1.880; 10.822]) were observed among those who were older than 30 year-old. (Tables 2 and 3).

**Table 2:** HIV-diagnosed MSM among all tested MSM in Rio de Janeiro, Brazil.

	Categories	No	Yes	UnadjustedRR [IC95%]; p-value	Adjusted*RR [IC95%]; p-value
	Overall	662 (83.48%)	131 (16.52%)		
Age	<30	398 (50.19%)	79 (9.96%)	reference	reference
	> = 30	264 (33.29%)	52 (6.56%)	0.992 [0.677; 1.455]; 0.969	0.949 [0.639; 1.410]; 0.797
Skin Color	White	294 (37.07%)	43 (5.42%)	reference	reference
	Non-white	365 (46.03%)	88 (11.10%)	1.648 [1.11; 2.449]; 0.013*	1.709 [1.145; 2.549]; 0.009**
Education	High School or Less	298 (37.58%)	68 (8.58%)	reference	reference
	Some College or Higher	359 (45.27%)	63 (7.94%)	0.769 [0.528; 1.120]; 0.171	0.811 [0.552; 1.194]; 0.289
12m hist. STD	no	591 (74.53%)	110 (13.87%)	reference	reference
	yes	71 (8.95%)	21 (2.65%)	1.589 [0.938; 2.694]; 0.085	1.629 [0.955; 2.778]; 0.074
12m hist. drug use	no	566 (71.37%)	114 (14.38%)	reference	reference
	yes	90 (11.35%)	16 (2.02%)	0.883 [0.500; 1.558]; 0.667	0.906 [0.508; 1.616]; 0.739
History of HIV test	Previously untested	537 (67.72%)	91 (11.48%)	reference	reference
	Previously tested	125 (15.76%)	40 (5.04%)	1.888 [1.241; 2.873]; 0.003*	1.954 [1.278; 2.986]; 0.002**
Strategy	Mobile unit	171 (21.56%)	43 (5.42%)	reference	reference
	NGO	491 (61.92%)	88 (11.10%)	0.713 [0.476; 1.068]; 0.100	0.787 [0.520; 1.191]; 0.258

* adjusted for: Skin Color, 12m hist. STD, and History of HIV test, when applicable

** p-value lower than 0.05.

**Table 3:** Linked to care among all HIV-diagnosed MSM in Rio de Janeiro, Brazil.

	Categories	No	Yes	UnadjustedRR [IC95%]; p-value	Adjusted*RR [IC95%]; p-value
	Overall	36 (27.48%)	95 (72.52%)		
Age	<30	27 (20.61%)	52 (39.69%)	reference	reference
	> = 30	9 (6.87%)	43 (32.82%)	2.603 [1.091; 6.211]; 0.031*	2.603 [1.091; 6.211]; 0.031**
Skin Color	White	16 (12.21%)	27 (20.61%)	reference	reference
	Non-white	20 (15.27%)	68 (51.91%)	2.015 [0.910; 4.459]; 0.084	2.137 [0.946; 4.828]; 0.068
Education	High School or Less	18 (13.74%)	50 (38.17%)	reference	reference
	Some College or Higher	18 (13.74%)	45 (34.35%)	0.900 [0.418; 1.939]; 0.788	0.723 [0.309; 1.692]; 0.455
12m hist. STD	No	31 (23.66%)	79 (60.31%)	reference	reference
	Yes	5 (3.82%)	16 (12.21%)	1.256 [0.424; 3.722]; 0.681	1.517 [0.490; 4.698]; 0.470
12m hist. drug use	No	31 (23.66%)	83 (63.36%)	reference	reference
	Yes	4 (3.05%)	12 (9.16%)	1.120 [0.336; 3.737]; 0.853	1.413 [0.409; 4.880]; 0.584
History of HIV test	Previously untested	26 (19.85%)	65 (49.62%)	reference	reference
	Previously tested	10 (7.63%)	30 (22.9%)	1.200 [0.514; 2.802]; 0.673	1.177 [0.490; 2.825]; 0.715
Strategy	Mobile unit	14 (10.69%)	29 (22.14%)	reference	reference
	NGO	22 (16.79%)	66 (50.38%)	1.448 [0.651; 3.223]; 0.364	1.381 [0.599; 3.181]; 0.449

* adjusted for: Age, and Skin Color, when applicable;

** p-value lower than 0.05.

In the bivariate analysis, individuals on cART had a lower mean CD4 count (460.26) than those who were not on cART (604.99) (p = 0.05 [Wilcoxon test]). In generalized linear models, there were no statistically significant predictors of use of cART among those MSM who were retained in care (S1 Table). Viral suppression among those who were on cART was suggestively lower (0.306 [0.089; 1.043]) for those MSM with a history of STD (S2 Table).

## Discussion

In this study, a community-based testing strategy in LGBT friendly settings was able to access hard-to-reach MSM and transwomen populations with a very high HIV prevalence. Alternative testing strategies, particularly those in partnership with the civil society, such as described in our study and in resource rich settings [17] are pivotal for the expansion of HIV testing among the most vulnerable. Among those individuals identified as HIV-infected, a low level of viral suppression after 12 months of follow up was observed. To our knowledge, this is the first study to evaluate the cascade of care continuum in the setting of a community based testing program for MSM and transwomen in a middle-income country.

Being non-white and having a prior HIV test were associated with an HIV diagnosis. For the Brazilian scenario, unlike in the United States of America, the association between HIV infection and race/skin color is not yet properly established. In most studies enrolling HIV-infected individuals as well as health outcomes studies enrolling the general population, this association loses its statistical significance when the model is controlled by sociodemographic variables such as education, income, and access to consumer goods. The variable race/skin color was only included in 1996 in Brazilian’s most important health information systems, as the ones used for vital statistics (Mortality Information System—SIM—and Live Birth Information System—SINASC), and, only in 2000, in the System for the Reporting of Notifiable Conditions (SINAN). Consequently, a more consistent analysis of the race/skin color -related vulnerability to HIV remains limited in our setting, and the results of the present work should be interpreted in light of these limitations. In relation to health services discrimination, young men self-declared as black reported more discriminative experiences in the context of hospitalization compared to white individuals [18].

Having a prior HIV test may represent a personal risk-reduction strategy or a consequence of a sustained high-risk behavior [18,19]. A large contemporary study investigating the relationship between HIV risk behavior and HIV testing among MSM in the United States found that MSM who screened repeatedly for HIV reported higher sexual risk behavior at study entry than MSM who only screened once and a repeated HIV testing was associated with an increase of risk behavior [20]. No prior studies in our setting have evaluated such association. It is crucial to better understand the dynamics of risk-taking behavior in order to adequately frame the counseling messages. Tailored prevention efforts should be developed and evaluated for MSM who repeatedly screen for HIV.

Young (< 30 year-old) individuals showed a lower likelihood for linkage and retention to HIV-care, in agreement with studies from high-income settings [21–24] and also from a clinical cohort in our city [25]. A significant and growing population of young MSM are acquiring and living with HIV in Brazil, with those aged 15 to 24 years accounting for the largest number of incident HIV infections in the country [6]. More than one in twenty MSM aged under 24 years old were HIV-infected in a time-location sampling survey [11]. Although late presentation to care was found to be associated with older ages [26–28], this is still a common finding among HIV-infected youth. In addition, suboptimal cART adherence and high risk sexual practices synergistically amplify HIV transmission [29,30]. Thus, the evaluation of novel strategies to enhance linkage and retention in care among young MSM is critical in our setting. When looking at our results vis a vis the overall Brazilian care continuum cascade [6], among the HIV-diagnosed individuals, a lower proportion of individuals were linked (72.5% vs. 91.1%, respectively; p < 0.01 [Chi squared]) and retained in care (68.7% vs. 76.1%, respectively; p = 0.04 [Chi squared]). Both cascades of care indicate that at each level, important percentages of those living with HIV fall out of the care continuum.

Robust benefits of early initiation of combination antiretroviral therapy (cART) in resource limited and resource rich settings have emerged in the past decade [31,32]. Moreover, preliminary results of the PARTNER Study and of the Opposites Attract Study have provided evidence that the effect of ART in preventing HIV sexual transmission is similarly present among MSM serodiscordant couples [33]. For the benefits of cART to be fully realized both at the individual and population-level, HIV infected individuals must be fully engaged in the “HIV continuum of care”. Incomplete engagement at any of the stages will compromise the impact of cART at the individual and community levels, leading to persistent HIV transmission and increased morbidity and mortality [22,34]. Data from developed and developing countries demonstrate that a substantial reduction in patient retention occurs between each stage of the HIV treatment continuum from diagnosis, initiation of cART, retention in care and viral suppression [35–39]. Stigma and discrimination may pose additional barriers to the utilization of health services leading to poorer health outcomes among specific groups of MSM and transwomen [40,41]. Moreover, stigma and discrimination, impose further delays in seeking care after HIV-infection diagnosis and poor ART adherence have been well documented in other settings [42–45] and may also explain the poorer linkage and retention to care in our population. Comparing again our results with the overall Brazilian care continuum cascade [6], despite a similar proportion of individuals having initiated cART (61.1% vs. 60.3%, respectively; p = 0.85 [Chi squared]), viral suppression among those under cART in our study was suggestively lower (62.5% vs. 71.8%, respectively; p = 0.06 [Chi squared]). Further studies are needed to assess the barriers related to retention to care and viral suppression in this population.

This manuscript has limitations. Foremost, the criteria we used for the cascade-construction allowed us to include a patient into a cascade-stage even if he did not necessarily pass through the immediately prior stage [46]. Additionally, given the small number of transwomen enrolled with a HIV-prevalence that was not statistically significant different from the MSM, we decided to aggregate in the analysis data from transwomen and MSM. However, potentially different risk factors may compromise the extrapolation of the findings for the transwomen population. The comparison between the present MSM cascade of HIV care and the overall Brazilian cascade is limited due to methodological reasons. Our cascade involves a smaller number of hard-to-reach individuals, with follow up restricted to the one-year after the HIV-positive test date. In contrast, the overall Brazilian cascade was constructed using only the information of individuals prescribed cART in the last 100 days of a given calendar year. Finally, individuals who have a private health insurance may have opted to use the private sector, which could potentially lead to an underestimation of linkage, retention to care and viral suppression levels due to underrepresentation of their CD4 and VL results in the national database. Nevertheless, it is unlikely that a considerable number of such individuals with access to private health-care would have been tested for HIV at the project locations, thus decreasing the likelihood of the underestimation of linkage, retention to care and viral suppression levels.

An important limitation of the current study is that it uses convenience sampling; all individuals had been tested for HIV. Given that lower socioeconomic status [47] and fewer years of education [47,48] are associated with low levels of HIV-testing, the sampling method used in our study may account for the observed higher level of education among the study participants. The convenience sampling method used in the current study was compared with the respondent driven sampling method in an earlier study [47]; the proportion of MSM with similar levels of education (some College, or higher), and prior test for HIV was higher using the convenience sampling method (53.2% vs. 36.4%, respectively; p < 0.001 [Chi squared]). However, in interpreting this difference the following two reasons should also be considered. First, in the five years between the studies (2008/2009 and 2013/2014), access to higher education (College) in Brazil has improved with 26.8% more people enrolled in presential graduation courses in 2014 when compared to 2009 [49]. Secondly, while Brito et al. [47] used self-reported information of prior HIV testing, the current study collected data from HIV-tested individuals using implemented community-based testing strategies.

## Conclusions

In conclusion, community-based testing strategy was effective in reaching a high-risk sample of MSM and a small sample of transwomen. Such strategy can be useful to identify high-risk individuals who can then be linked to prevention and care services. Despite the universal care coverage and the test-and-treat policy adopted in Brazil, our results indicate that strategies to increase linkage to care and prompt cART initiation are critically needed. Interventions for promoting engagement in HIV-care targeting non-white and young MSM should be prioritized.

## Supporting Information

S1 Table On cART among those MSM Retained in care in Rio de Janeiro, Brazil. (DOCX)

S2 Table Virally suppressed among those MSM on cART in Rio de Janeiro, Brazil. (DOCX)
