# Supplementary material for: The Men Who Have Sex with Men HIV Care Cascade in Rio de Janeiro, Brazil
Source: PLoS One. 2016 Jun 14;11(6):e0157309. doi: 10.1371/journal.pone.0157309 (PMC4907447; doi:10.1371/journal.pone.0157309)
Supplement: S1 Table — (DOCX) [file pone.0157309.s001.docx]

**S1 Table. On cART among those MSM Retained in care in Rio de Janeiro, Brazil.**

|  | Categories | No | Yes | Unadjusted  RR [IC95%]; p-value | Adjusted*  RR [IC95%]; p-value |
| --- | --- | --- | --- | --- | --- |
|  | Overall | 10 (11.11%) | 80 (88.89%) |  |  |
| Age | <30 | 6 (6.67%) | 43 (47.78%) | reference | reference |
|  | >=30 | 4 (4.44%) | 37 (41.11%) | 1.291 [0.338; 4.925]; 0.709 | 1.812 [0.458; 7.175]; 0.397 |
| Skin Color | White | 2 (2.22%) | 23 (25.56%) | reference | reference |
|  | Non-white | 8 (8.89%) | 57 (63.33%) | 0.62 [0.122; 3.141]; 0.563 | 0.578 [0.110; 3.031]; 0.517 |
| Education | High School or Less | 5 (5.56%) | 41 (45.56%) | reference | reference |
|  | Some College or Higher | 5 (5.56%) | 39 (43.33%) | 0.951 [0.255; 3.542]; 0.941 | 1.409 [0.364; 5.461]; 0.620 |
| 12m hist. STD | no | 8 (8.89%) | 67 (74.44%) | reference | reference |
|  | yes | 2 (2.22%) | 13 (14.44%) | 0.776 [0.148; 4.080]; 0.765 | 0.711 [0.127; 3.981]; 0.698 |
| 12m hist. drug use | no | 8 (8.89%) | 70 (77.78%) | reference | reference |
|  | yes | 2 (2.22%) | 10 (11.11%) | 0.571 [0.106; 3.082]; 0.515 | 0.609 [0.107; 3.474]; 0.576 |
| History of HIV test | Previously untested | 5 (5.56%) | 57 (63.33%) | reference | reference |
|  | Previously tested | 5 (5.56%) | 23 (25.56%) | 0.404 [0.107; 1.527]; 0.181 | 0.432 [0.110; 1.704]; 0.231 |
| Strategy | Mobile unit | 0 (0%) | 27 (30%) | reference | reference |
|  | NGO | 10 (11.11%) | 53 (58.89%) | 0 [0; Inf]; 0.993 | 0 [0; Inf]; 0.993 |

* adjusted for: Strategy, when applicable.
