# Supplementary material for: The Men Who Have Sex with Men HIV Care Cascade in Rio de Janeiro, Brazil
Source: PLoS One. 2016 Jun 14;11(6):e0157309. doi: 10.1371/journal.pone.0157309 (PMC4907447; doi:10.1371/journal.pone.0157309)
Supplement: S2 Table — (DOCX) [file pone.0157309.s002.docx]

**S2 Table. Virally suppressed among those MSM on cART in Rio de Janeiro, Brazil.**

|  | Categories | No | Yes | Unadjusted  RR [IC95%]; p-value | Adjusted*  RR [IC95%]; p-value |
| --- | --- | --- | --- | --- | --- |
|  | Overall | 30 (37.5%) | 50 (62.5%) |  |  |
| Age | <30 | 17 (21.25%) | 26 (32.50%) | reference | reference |
|  | >=30 | 13 (16.25%) | 24 (30.00%) | 1.002 [0.387; 2.597]; 0.996 | 0.692 [0.255; 1.873]; 0.468 |
| Skin Color | White | 10 (12.50%) | 13 (16.25%) | reference | reference |
|  | Non-white | 20 (25.00%) | 37 (46.25%) | 1.423 [0.530; 3.821]; 0.484 | 1.419 [0.516; 3.906]; 0.498 |
| Education | High School or Less | 18 (22.50%) | 23 (28.75%) | reference | reference |
|  | Some College or Higher | 12 (15.00%) | 27 (33.75%) | 1.761 [0.703; 4.410]; 0.227 | 1.483 [0.571; 3.847]; 0.418 |
| 12m hist. STD | no | 22 (27.50%) | 45 (56.25%) | reference | reference |
|  | yes | 8 (10.00%) | 5 (6.25%) | 0.306 [0.089; 1.043]; 0.058** | 0.306 [0.089; 1.043]; 0.058** |
| 12m hist. drug use | no | 25 (31.25%) | 45 (56.25%) | reference | reference |
|  | yes | 5 (6.25%) | 5 (6.25%) | 0.556 [0.147; 2.106]; 0.387 | 0.649 [0.163; 2.585]; 0.540 |
| History of HIV test | Previously untested | 22 (27.50%) | 35 (43.75%) | reference | reference |
|  | Previously tested | 8 (10.00%) | 15 (18.75%) | 1.179 [0.429; 3.237]; 0.750 | 1.319 [0.462; 3.768]; 0.605 |
| Strategy | Mobile unit | 13 (16.25%) | 14 (17.50%) | reference | reference |
|  | NGO | 17 (21.25%) | 36 (45.00%) | 1.966 [0.760; 5.085]; 0.163 | 1.948 [0.736; 5.156]; 0.179 |

* adjusted for: 12m hist. STD, when applicable; ** p-value lower than 0.06.
